# Synthesis and Characterization of Wooden Magnetic Activated Carbon Fibers with Hierarchical Pore Structures

**DOI:** 10.3390/polym10040435

**Published:** 2018-04-13

**Authors:** Dongna Li, Jianing Li, Biyun Ren, Tongtong Li, Xiaojun Ma

**Affiliations:** 1 Ministry of AgricultureKey Laboratory of Biology and Genetic Resource Utilization of Rubber Tree/State Key Laboratory Breeding Base of Cultivation & Physiology for Tropical Crops, Rubber Research Institute, Chinese Academy of Tropical Agricultural Sciences, Danzhou 571737, China; lidnqiaoh@126.com (D.L.); ljn206@163.com (J.L.); tongxinltt@163.com (T.L.); 2 College of Packaging & Printing Engineering, Tianjin University of Science & Technology, Tianjin 300222, China; byren2-c@my.cityu.edu.hk

**Keywords:** magnetic iron oxide nanoparticles, wooden activated carbon fiber, biocompatible, adsorption, magnetic properties

## Abstract

Wooden magnetic activated carbon fibers (WMACFs) with hierarchical pore structures were obtained by adding magnetic iron oxide (Fe_3_O_4_) nanoparticles into the liquefied wood. The structures and properties of WMACFs were analyzed by scanning electronmicroscopy (SEM), X-ray diffraction (XRD), Fourier transform infrared spectroscopy (FTIR), N_2_ adsorption, and vibrating sample magnetometer (VSM). The results showed that WMACFs had high Brunauer-Emmett-Teller (BET) surface area (1578 m^2^/g) and total pore volume (0.929 cm^3^/g), of which 45% was the contribution of small mesopores of 2–3 nm. It is believed that Fe_3_O_4_ nanoparticles play an important role in the formation of hierarchical pores. With the Fe_3_O_4_ content increasing, the yield rate of WMACFs decreased, and the Fe_3_O_4_ crystal plane diffraction peaks and characteristic adsorption peaks were obviously observed. At the same time, it was also found that WMACFs had favorable magnetic properties when the Fe_3_O_4_ content was above 1.5%. As a result, WMACFs could be a promising candidate for high efficiency, low cost, and convenient separation for the magnetic field.

## 1. Introduction

Activated carbon fibers (ACFs), which have uniform slit-shaped micropores and great surface area, have played a major role in adsorption technology over the last few years [1]. However, ACFs are microporous with pore diameter ranging from 0.3 to 0.5 nm [2], resulting in challenges for applying ACFs in some areas, especially those that require adsorption of bulky molecules from solutions and macromolecular reactions with catalyst supports evolved. Recently, ACFs with hierarchical pore structures have attracted much attention because of their excellent adsorptive properties based on highly developed multi-porous structures [3,4,5]. Various methods have been carried out to prepare ACFs with hierarchical pore structures. For example, previous research has shown that wooden activated carbon fibers (WACFs) with highly developed mesopores have been successfully prepared by steam activation with the addition of wood charcoal, resulting in the specific Brunauer-Emmett-Teller surface area (*S*_BET_) and the ratio of the mesopore volume to the total pore volume (MP-ratio) of ACFs increasing significantly [6,7]. 

Moreover, the used ACFs often suffer from serious problems of separation in liquid-solid phase processes and cause secondary pollution to the environment [8]. Magnetic technology makes it possible to effectively separate and recover the spent ACFs through a simple magnetic process and exhibits excellent ability to separate nanosized materials with many advantages, including easy operation and low cost [9]. Fe_3_O_4_ nanoparticles are the most popular and commonly used magnetic source in magnetic separation due to its superior magnetic performance, low toxicity, and easy preparation [10]. Li et al. [11] designed and prepared a unique, multifunctional Fe_3_O_4_-activated carbon-sodium alginate composite absorbent (MSA-AC) that extracted dye from aqueous solutions. The results revealed that the MSA-AC has a potential application in wastewater treatment and in the development of composite absorbent that is simple and fast to prepare, cost-effective, and environmentally friendly. Shi et al. [12] prepared carbon–iron composites from waste cation exchange resin through NaOH activation. The composite, synthesized at 800 °C, could effectively remove diethyl phthalate, bisphenol A, and malachite green from aqueous solutions. Yang et al. [13] synthesized magnetic Fe_3_O_4_-activated carbon nanocomposite from rice husk based activated carbon. The system demonstrated perfect magnetic separation performance and a high adsorption capacity of 321 mg/g for methylene blue (MB) from aqueous solutions. It is expected that the obtained magnetic materials can be used as potential sorbents for the removal of various toxic pollutants from wastewater. In recent years, scholars have explored the introduction of magnetic nanoparticles into ACFs and have become more focused on physically mixing ACFs with metallic oxide before carbonization and activation to obtain ACFs with hierarchical pore structures through the catalytic activation method [14,15]. It is expected that Fe_3_O_4_ nanoparticles as additives will play an important role in the multi-pore formation of ACFs. Currently, most methods synthesize Fe_3_O_4_ in a carbon matrix using activated carbon materials as the carbon source, such as using the impregnating method in Fe^3+^ solution [16]. This, however, only loads the magnetic nanoparticles on the surface of the ACFs, and the supported nanoparticles easily fall off, resulting in the decrease of the adsorption capacity and reuse times after adsorption saturation. Very few studies have been done that combine Fe_3_O_4_ nanoparticles with WACFs. 

Based on our previous work [17,18], using Fe_3_O_4_ nanoparticles as an additive, wooden magnetic activated carbon fibers (WMACFs) from Chinese fir (*Cunninghamia lanceolata*) were prepared by phenol liquefaction, melt spinning, curing, and activation using steam. The morphological, chemical, and microcrystalline structures of the synthesized WMACFs were characterized using a wide array of analytical methods. In addition, in order to understand the relationship between Fe_3_O_4_ contents, pore size distribution, and magnetic properties, the WMACFs were studied. The effects of different Fe_3_O_4_ contents on the specific surface area and magnetic properties were investigated in detail by N_2_ adsorption and vibrating sample magnetometer (VSM). This study effectively solves the disadvantages of the impregnating method and can obviously increase reuse times. It also provides reference value for further research.

## 2. Materials and Methods

### 2.1. Samples

**Figure 1:**
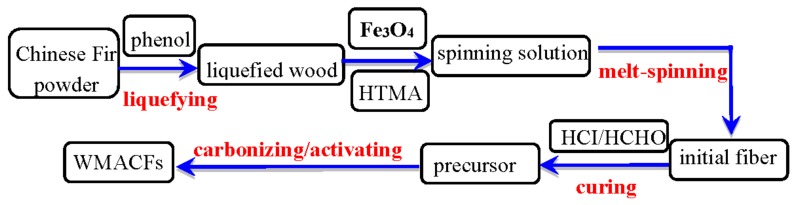
Schematic of the production process of the wooden magnetic activated carbon fibers (WMACFs). HTMA: hexamethylenetetramine; HCI: hydrochloric acid; and HCHO: formaldehyde.

Chinese fir (*Cunninghamia lanceolata*) was firstly ground and screened to a particle size of 60–80 meshes to prepare the precursor fibers through a series of processes, including liquefaction, adding nano-Fe_3_O_4_ (Fe content ≥95%, relative density was 5.18 g/cm^3^, melting point was 1594 °C), melt-spinning, and curing in accordance with previous studies [19,20]. The precursors were put in a tube furnace, and the activation process was conducted with a temperature program from room temperature to the final activation temperature (800 °C) using a heating rate of 5 °C/min under N_2_ (200 cm^3^/min). The precursors were held isothermally for 40 min under a steam flow of 8 g/min and then cooled to room temperature to obtain the WMACFs [21]. Various contents (0–2.5 wt %) of Fe_3_O_4_ were used to investigate its influence on the WMACFs. The schematic of the production process is shown in Figure 1.

### 2.2. Characterizations

The surface morphologies of the WMACFs were examined using a SEM (NOVA Nano SEM430, FEI, Hillsboro, OR, USA) with an acceleration voltage of 10 keV. 

The X-ray diffraction (XRD) analyses of the WMACFs were carried out at room temperature on a power X-ray diffractometer (D/max-2500, Rigaku, Tokyo, Japan) using Cu Kα radiation (wavelength was 0.154 nm, powdered samples). The XRD analysis conditions were as follows: scanning range of 5°–60°, scanning rate of 2°/min at 40 kV and 100 mA.

In order to examine the differences between the microcrystalline structures of the WMACFs at various Fe_3_O_4_ contents, the apparent value of the planar size (*L*a_(110)_), the bulk thickness (*L*c_(002)_) of the graphite sheet layer, the average crystallite size (*D*), and the layer spacing *d*_002_ were calculated using the Scherrer and Bragg formulas [22,23]. The formulas were as follows:

*L*c_(002)_, *D* = 0.89λ/βcosθ (1)

*L*a_(110)_ = 1.84λ/βcosθ (2)

*d*_002_ = λ/2sinθ (3)

where λ is the wavelength of the X-ray (0.154 nm); θ is the Bragg angle of (002), (100), and (311) peaks (°); and β is the half-height width of (002), (100), and (311) peaks (rad).

The graphitization degree of the WMACFs can be calculated by the layer spacing *d*_002_ [24]. The simplified formula is

*g* = (0.3440 − *d*_002_)/(0.3440 − 0.3554) (4)

where *g* is the graphitization degree (%); 0.3440 is the layer spacing of not completely graphited carbon material (nm); 0.3354 is the layer spacing of the ideal graphite crystal (nm); and *d*_002_ is the layer spacing (nm).

The chemical characterization of the functional groups was detected using pressed potassium bromide (KBr) pellets containing 5% of the sample by Fourier-transform infrared spectrometry (Nicolet-6700, Thermo electron, Waltham, MA, USA) in the scanning range of 4000–500 cm^−1^.

The specific surface area and the porosity of the samples were determined by N_2_ adsorption-desorption isotherm measured at 77 K in a Micromeritics ASAP-2020 apparatus (Micromeritics, Norcross, GA, USA. Before analysis, the samples were degassed at 350 °C for 2 h. The S_BET_ was calculated by the Brunauer-Emmett-Teller (BET) method using the N_2_ adsorption isotherm data. The total pore volume (*V*_tot_) was evaluated by converting the amount of N_2_ adsorbed at a relative pressure of 0.995 to the volume of the liquid adsorbate. The micropore area (*S*_micro_) and micropore volume (*V*_micro_) were obtained by t-plot method [25]. The mesopore area (*S*_meso_) and mesopore volume (*V*_meso_) were calculated by Barrett-Joyner-Halenda (BJH) method [26]. Pore size distributions were calculated using the density functional theory (DFT) Plus software (provided by Micromeritics Instrument Corporation (Georgia, USA), which was based on calculated adsorption isotherms for pores of different sizes [27]. This program performs an inversion of the integral equation for the overall adsorption isotherm with respect to pore size distributions.

The yield rate η of the WMACFs can be expressed as:

η = *m*/*m*_0_ × 100% (5)

where *m*_0_ and *m* are the masses of the specimen before and after activation, respectively. 

The magnetic properties of the WMACFs were measured with a Lake Shore 7304 VSM (Lakeshore, Columbus, MS, USA) at room temperature.

## 3. Results and Discussion

### 3.1. Morphological Characteristics of the WMACFs

**Figure 2:**
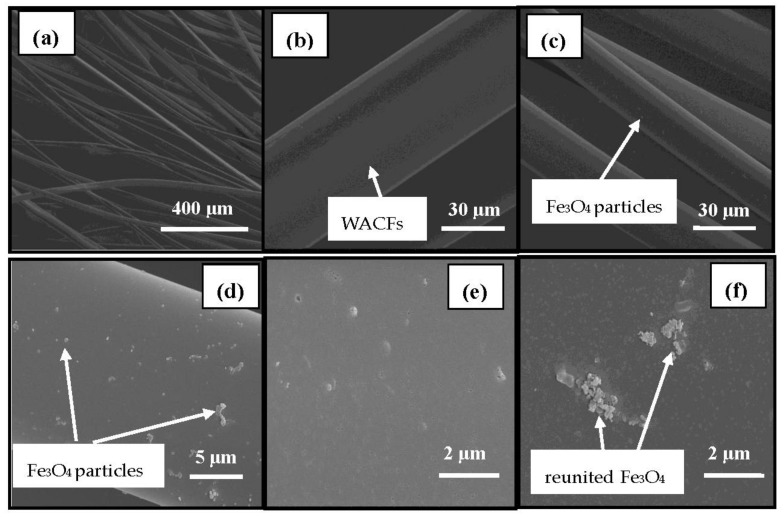
Surface SEM photographs of wooden activated carbon fibers (WACFs) (**a**,**b**) and WMACFs (2.5% Fe_3_O_4_ content) (**c**–**f**).

The SEM images of the surfaces of the WACFs and the WMACFs are shown in Figure 2a–f. It is obvious that the WACFs (Figure 2a,b) have a smooth surface and uniform thickness without surface deposits, and that they maintain porous structures on the inner and outer surface. Figure 2c–f shows that Fe_3_O_4_ nanoparticles were deposited on almost every WMCAF with non-uniform distribution, and the Fe_3_O_4_particles’ agglomeration and pores can be clearly observed.

**Figure 3:**
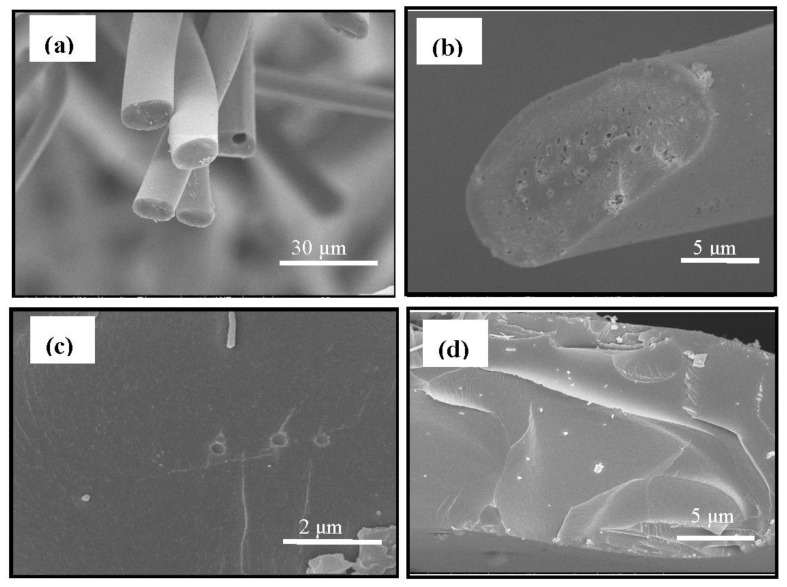
Cross-sectional SEM photographs of the WACFs (**a**,**b**) and WMACFs (2.5% Fe_3_O_4_ content) (**c**,**d**).

Figure 3 shows a SEM image of a cross-section of the WACFs (Figure 3a,b) and WMACFs (Figure 3c,d). Figure 3a,b shows that the cross-section of the WACFs was round or elliptical, and some unevenly distributed pores were possibly caused by the number of escaping non-carbon atoms, which increased during the carbonization and activation process. Figure 3c,d shows that some Fe_3_O_4_ particles were present. It was found that the granular Fe_3_O_4_ was also deposited onto the cross-section of the WMACFs during the reaction.

### 3.2. XRD Analysis of the WMACFs

**Figure 4:**
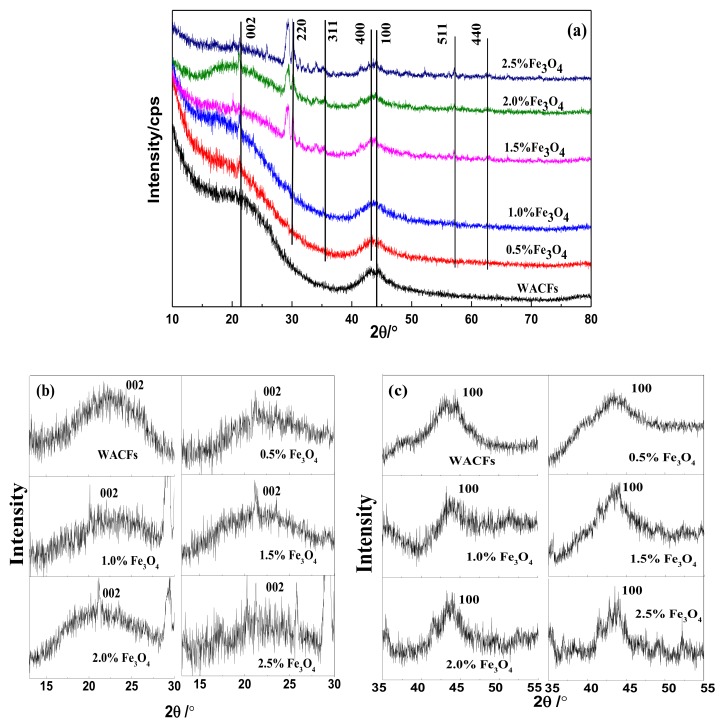
(**a**) XRD pattern of the WACFs and WMACFs at various Fe_3_O_4_ contents; (**b**,**c**) Local magnification curve of (002) and (100) peaks at various Fe_3_O_4_ contents.

Figure 4a showed the XRD patterns of the WACFs and WMACFs at various Fe_3_O_4_ contents. The observed peaks at 2θ = 21.3° and 43.9° were (002) and (100) peaks of disordered graphite microcrystals in the WACFs, respectively [28]. The occurrence of the dominant peaks at 2θ = 30.3°, 35.3°, 43.5°, 57°, and 62.7° could correspond to (220), (311), (400), (511), and (440) crystal planes of a pure Fe_3_O_4_ with a spinal structure (JCPDS file (PDF No. 65-3107)) [29]. All of the above were the characteristic diffraction peaks of Fe_3_O_4_ [30,31], indicating that Fe_3_O_4_ had been introduced into the WACFs. As the nano-Fe_3_O_4_ content was low, its peaks’ intensities were relatively weak, especially when the content was 0.5% and 1%. With increased Fe_3_O_4_ content, the diffraction peaks were narrowed and became more prominent. It was significant that the changes of (220) and (400) peaks, which indicated the crystallization degree of Fe_3_O_4,_ were enhanced. In addition, for the samples of 1.5–2.5% Fe_3_O_4_ content, the diffraction peak at 2θ = 28°–30.3° had a great change compared with other samples, displaying the double peaks phenomenon. This was probably because under the high-temperature activation process, with the increase of the Fe_3_O_4_ content, the structure of the samples had undergone a phase transformation from single-phase to two-phase, maintaining a two-phase coexistence state.

As shown from the XRD structure parameters (Table 1), *d*_002_, *L*_c(002)_, *L*_a(100)_, and g were calculated by Formulas (1)–(4), respectively. β of (002) and (100) peaks was clearly seen in Figure 4b,c. From the calculation results, the increase of the Fe_3_O_4_ content from 0% to 2.5% led to an obvious decrease in *d*_002_ from 0.4931 to 0.3237 nm. It can be concluded that the Fe_3_O_4_ content had a significant influence on the *d*_002._ In addition, the bulk thickness (*L*_c(002)_) and the apparent layer-plane length parallel to the fibers axis (*L*_a(100)_) increased from 0.9561 to 1.064 nm and from 3.232 to 4.303 nm, respectively. The *L*_c_/*d*_002_ and g value corresponding to the change degree of graphitization structure also increased. The overall increase of *L*_c(002)_ was due to the fact that a large number of substances of aromatic structure that formed during the carbonization and activation process gradually changed into graphite-like microcrystalite structures of multilayer stacks. Correspondingly, the increase of *L*_a(100)_ indicated that a more regular and ordered carbon structure had been formed with the increased Fe_3_O_4_ content.

Table 2 shows the nanoparticle size of Fe_3_O_4_ as calculated by Formula (1). It can be observed that the average crystallite size of Fe_3_O_4_ was rather small, in the range of 10–19 nm. The reasons for the smaller particle size might be that the Fe_3_O_4_ particles were evenly and firmly dispersed on the surface of the WMACFs, without aggregation, providing a superior condition for the adsorption and separation.

### 3.3. FTIR Analysis of the WMACFs

**Figure 5:**
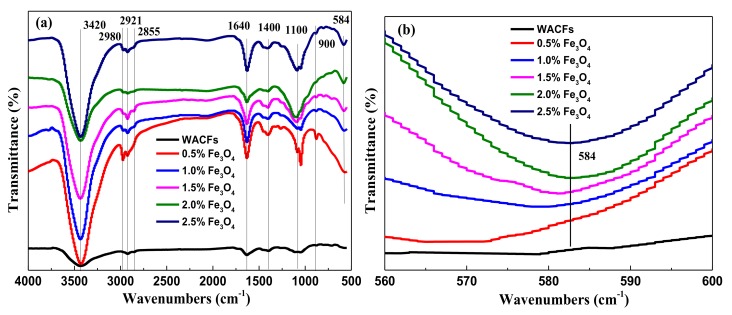
(**a**) FTIR of the WACFs and WMACFs at various Fe_3_O_4_ contents; (**b**) Local magnification curve of 584 cm^−1^ vibrationsat various Fe_3_O_4_ contents.

Figure 5a shows the FTIR spectra of the WACFs and WMACFs at various Fe_3_O_4_ contents, respectively. The position and shape of the band at 3420 cm^−1^ (–OH) were compatible with the involvement of hydrogen-bonded hydroxyl groups. The band at 3420 cm^−1^ was slightly broader towards the lower wavelengths, suggesting that some OH-ether hydrogen bonds were present. Weak bands at 1400 cm^−1^ were the stretching vibration of non-associated –OH. The 1640 cm^−1^ band indicated that a variety of C=C bonds existed besides those in aromatic rings. Both the WACFs and WMACFs showed weak adsorption peaks at 2980, 2921, and 2855 cm^−1^, which were due to the stretching vibration of C–H [5,32]. The broad band at 1100 cm^−1^ was the stretching vibration of C–O–C and C–O–H, whose vibrations became flat. The adsorption peaks at 900 cm^−1^ (C–C) decreased. The new band at 584 cm^−1^ (Figure 5b) could be attributed to the stretching vibrations of Fe–O [10]. It proved that Fe_3_O_4_ was well-deposited on the WMACFs. As the nano-Fe_3_O_4_ content was low, its adsorption peak was relatively weak.

### 3.4. Adsorption Characteristics, Specific Surface Area, and Pore Distribution of the WMACFs

**Figure 6:**
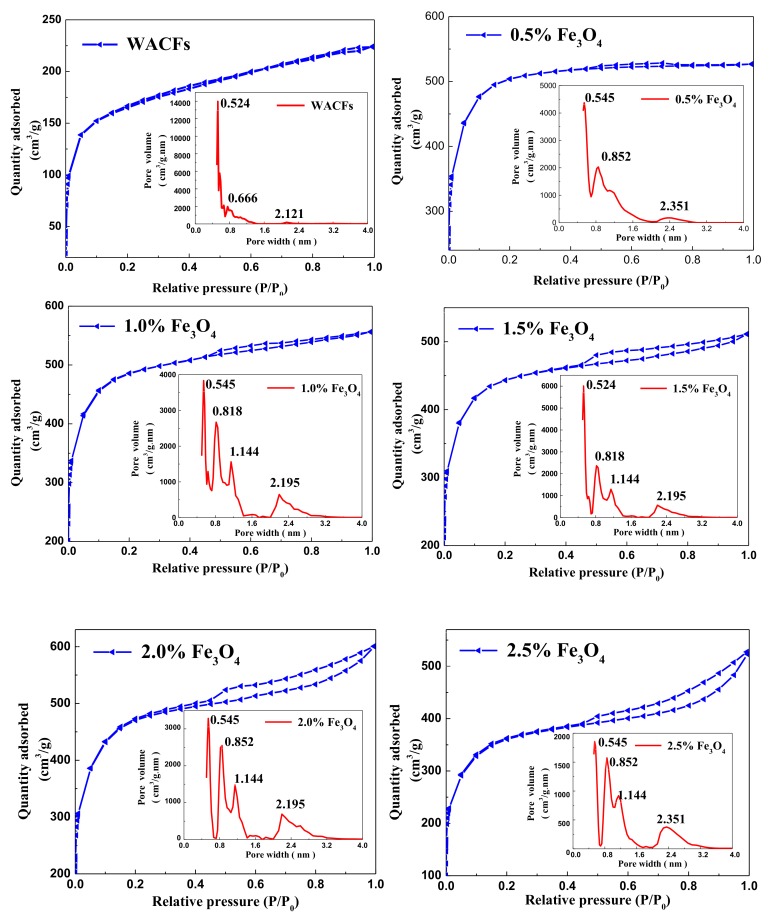
Nitrogen adsorption-desorption isotherms and pores size distribution of the WACFs and WMACFs.

Figure 6 showed the N_2_ adsorption–desorption isotherms and pores size distribution of the WACFs and WMACFs, respectively. The adsorption isotherm of the WACFs and WMACFs of 0.5% Fe_3_O_4_ content was typical type I, based on the International Union of Pure and Applied Chemistry (IUPAC) classification where microporous adsorption was dominating, also called the Langmuir isother [33,34]. The isotherm profiles of the WMACFs (except for 0.5% Fe_3_O_4_ content) belonged to typical type IV in which an adsorption/desorption hysteresis loop was visible, which can be ascribed to a defining characteristic of mesoporosity [35,36]. This meant that the Fe_3_O_4_ content had a great effect on the development of mesoporosity.

As shown in Figure 6, the pore size distribution of the WMACFs can be divided into two prominent parts. The first part demonstrated a few peaks that were sharp and occurred at ca. 0.5 and 1.2 nm. The second part was in the range between 2.0 and 3.0 nm. This demonstrated that the WMACFs were a combination of micropores and mesopores with hierarchical pore structures. However, the microporous structure was still dominant.

**Figure 7:**
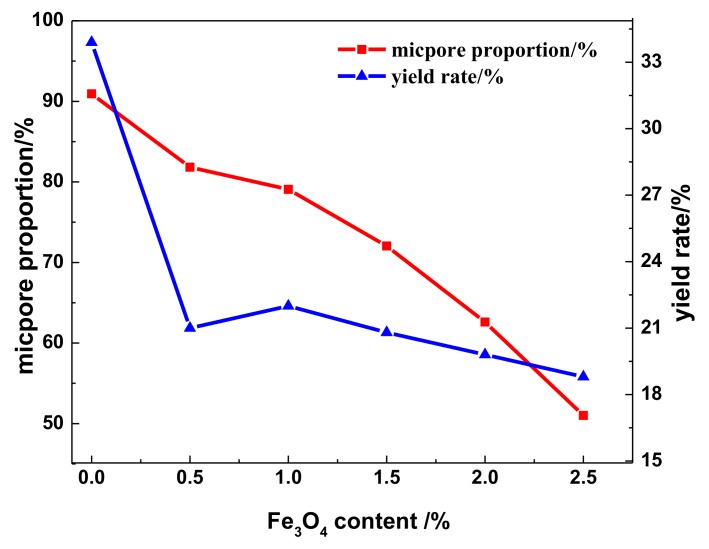
Micropore proportion and yield rate of the WMACFs.

The textural parameters of the WMACFs are listed in Table 3. As the Fe_3_O_4_ content was increased from 0% to 2.5%, the *S*_BET_, *S*_micro_, and *V*_micro_ all showed decreasing trends; the *V*_tot_ gradually decreased and then increased. However, the *S*_meso_, *V*_meso_, and MP-ratio were increased. Especially when the Fe_3_O_4_ content was increased from 0% to 0.5%, both the *S*_meso_ and *V*_meso_ increased by 68.47% and 71.83%, respectively. This indicated that after adding Fe_3_O_4_, the ultra-micropores were enlarged, and most of them were further developed to mesopores, especially at a Fe_3_O_4_ content of 0.5%. However, when the Fe_3_O_4_ content was too high, the decrease of *S*_BET_ suggested the formation of mesopores with hierarchical pore structures. As shown in Figure 7, the micropore proportion in the total pore volume and yield rate decreased from 90.93% and 33.9% to 51.02% and 18.8%, respectively. The yield rate of the WMACFs via activation had an obvious decrease due to the fact that more carbon was eroded to develop the porosity. This was also related to the pore-forming and pore-expanding function of Fe_3_O_4_.

### 3.5. VSM Analysis of the WMACFs

**Figure 8:**
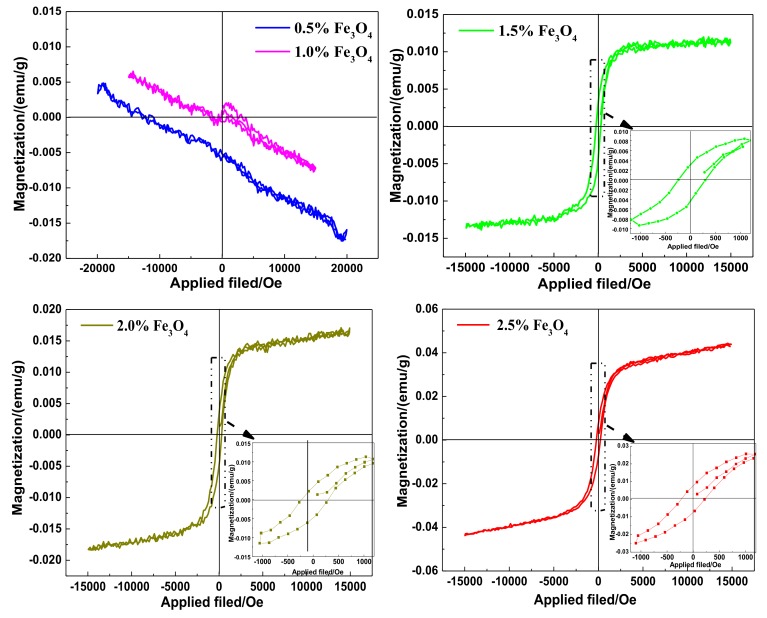
The magnetic hysteresis loop of the WMACFs.

Figure 8 shows the magnetic hysteresis loop of the WMACFs. When the Fe_3_O_4_ content increased from 0.5% to 1.5%, the WMACFs responded weakly to an external magnetic field. This is probably because the lower Fe_3_O_4_ content was not enough to make the WMACFs possess magnetic properties. Whereas when the Fe_3_O_4_ content was increased from 1.5% to 2.5%, the very weak hysteresis revealed the resultant magnetic nanoparticles were nearly of magnetic properties with a saturation magnetization from 0.0129 to 0.0440 emu/g. This meant that the Fe_3_O_4_ content was a key factor influencing the magnetic properties of the WMACFs. Because of the low nano-Fe_3_O_4_ content and small nanoparticle size, the saturation magnetization was relatively small. However, the samples still had adequate magnetization to be easily and quickly separated from the complex sample. 

Table 4 shows the residual magnetization, saturation magnetization, and coercive force of the WMACFs. With increased Fe_3_O_4_ content, the saturation magnetization increased, which was probably due to the fact that the greater the Fe_3_O_4_ content, the more obvious the magnetism. The residual magnetization increased from 0.0037 to 0.0075 emu/g. When the Fe_3_O_4_ content was 1.5%, the coercive force reached its maximum, which was 270.4 Oe. The samples with Fe_3_O_4_ contents of 1.5% to 2.5% had certain residual magnetization and coercive force, which was due to the partial agglomeration of the Fe_3_O_4_ nanoparticles during the carbonization and activation process. 

## 4. Conclusions

In this study, WMACFs were successfully synthesized by phenol liquefaction, adding nano-Fe_3_O_4_, and melt-spinning, as well as curing and activation. Nano-Fe_3_O_4_ existed on the surface and cross-sections of almost every WMACF. With increased Fe_3_O_4_content, the yield rate of the WMACFs decreased, and the Fe_3_O_4_crystal plane diffraction peaks were obviously heightened. The characteristic adsorption peaks of Fe–O emerged at 594 cm^−1^ on the infrared spectrum. The WMACFs had a narrow distribution of micropore diameters (0.5–1.2 nm) and mesopore diameters (2.0–3.0 nm). Due to the pore-forming and pore-expanding function of Fe_3_O_4_, higher *S*_BET_ and more developed pore structures were achieved. When the Fe_3_O_4_content was more than 1.5%, it had favorable magnetic properties, which provided a reference value for the recovery of the WMACFs after adsorption saturation. The practical application of the WMACFs was limited because of the low Fe_3_O_4_ content, but this provides a new direction to explore wooden activated carbon fiber materials with highly developed multi-porous structures and magnetic properties for adsorption separation. It is expected to be used in supercapacitor electronic materials, wastewater treatment, and air purification, such as the adsorption of formaldehyde, as well as in carrier materials of photocatalyst due to its hierarchical pore structures, magnetic properties, and characteristics of doping metal oxide. 

## Figures and Tables

**Table 1:** XRD structure parameters for the WMACFs.

Fe_3_O_4_ Content	*d*_(002)_/nm	*L*c_(002)_/nm	*L*a_(100)_/nm	*L*c/*d*_(002)_	*g*/%
0%	0.4931	0.9561	3.232	1.939	−17.34
0.5%	0.5657	0.9350	3.027	1.653	−25.78
1.0%	0.5491	0.9554	3.625	1.739	−23.86
1.5%	0.4635	0.9748	3.809	2.103	−13.89
2.0%	0.4553	1.003	4.239	2.203	−12.94
2.5%	0.3237	1.064	4.303	3.287	2.36

**Table 2:** Relationship between the Fe_3_O_4_ content and the average crystallite size of Fe_3_O_4_.

**Fe_3_O_4_ Content**	0.5%	1%	1.5%	2%	2.5%
**Average Crystallite Size**	18.73 nm	14.22 nm	11.13 nm	11.45 nm	10.58 nm

**Table 3:** The textural parameters of the WMACFs from the adsorption isotherms of nitrogen ^1^.

Fe_3_O_4_ Content	*S*_BET_ (m^2^/g)	*S*_micro_ (m^2^/g)	*S*_meso_ (m^2^/g)	*V*_to__t_ (m^2^/g)	*V*_micro_ (m^2^/g)	*V*_meso_ (m^2^/g)	MP-Ratio (%)
0%	1173	1090	64	0.551	0.501	0.040	7
0.5%	1894	1667	203	0.858	0.702	0.142	17
1.0%	1816	1550	203	0.836	0.661	0.154	18
1.5%	1657	1380	241	0.737	0.531	0.181	25
2.0%	1716	1377	299	0.896	0.561	0.300	33
2.5%	1578	1206	322	0.929	0.474	0.415	45

^1^ *S*_BET_, Brunauer-Emmett-Teller surface area; *S*_micro_, micropore surface area; *S*_meso_, mesopore surface area; *V*_tot_, total pore volume; *V*_micro_, micropore volume; *V*_meso_, mesopore volume; and MP-ratio = (*V*_meso_/*V*_tot_) × 100%.

**Table 4:** Residual magnetization, saturation magnetization, and coercive force of the WMACFs.

Fe_3_O_4_ Content	0.5%	1.0%	1.5%	2.0%	2.5%
**Residual Magnetization (emu/g)**	-	-	0.0037	0.0041	0.0075
**Saturation Magnetization (emu/g)**	-	-	0.0129	0.0177	0.0440
**Coercive Force (Oe)**	-	-	270.4	261.3	224.1
